# Changes in poly(A) tail length dynamics from the loss of the circadian deadenylase Nocturnin

**DOI:** 10.1038/srep17059

**Published:** 2015-11-20

**Authors:** Shihoko Kojima, Kerry L. Gendreau, Elaine L. Sher-Chen, Peng Gao, Carla B. Green

**Affiliations:** 1 Department of Neuroscience, University of Texas Southwestern Medical Center, Dallas, TX, USA, 75390-9111; 2 Department of Biological Sciences, Virginia Bioinformatics Institute, Virginia Tech, Blacksburg, VA, USA, 24061

## Abstract

mRNA poly(A) tails are important for mRNA stability and translation, and enzymes that regulate the poly(A) tail length significantly impact protein profiles. There are eleven putative deadenylases in mammals, and it is thought that each targets specific transcripts, although this has not been clearly demonstrated. Nocturnin (NOC) is a unique deadenylase with robustly rhythmic expression and loss of *Noc* in mice (*Noc* KO) results in resistance to diet-induced obesity. In an attempt to identify target transcripts of NOC, we performed “poly(A)denylome” analysis, a method that measures poly(A) tail length of transcripts in a global manner, and identified 213 transcripts that have extended poly(A) tails in *Noc* KO liver. These transcripts share unexpected characteristics: they are short in length, have long half-lives, are actively translated, and gene ontology analyses revealed that they are enriched in functions in ribosome and oxidative phosphorylation pathways. However, most of these transcripts do not exhibit rhythmicity in poly(A) tail length or steady-state mRNA level, despite *Noc’s* robust rhythmicity. Therefore, even though the poly(A) tail length dynamics seen between genotypes may not result from direct NOC deadenylase activity, these data suggest that NOC exerts strong effects on physiology through direct and indirect control of target mRNAs.

Poly(A) tails are hallmarks of most eukaryotic mRNAs, serving to protect the mRNA from degradation and promote circularization of the RNA to allow efficient translation initiation^1^. Changes in poly(A) length occur throughout the life of the mRNA, and long poly(A) tails of ~150–250 nt are initially acquired during the 3′ end processing of the nascent transcripts in the nucleus. After transcripts are translocated into the cytoplasm, they subsequently become shortened by deadenylases, a group of ribonucleases specific for homopolymeric tracts of adenosines^1^. Deadenylation is a rate-limiting step for mRNA degradation that occurs in both mRNA- and context- specific manners, and can result in RNA de-stabilization or translational silencing^2^.

There are 11 putative deadenylases currently identified in mammals based on their sequence homology, and most of them have been shown to play important roles in regulating diverse processes, ranging from general viability of organisms to bone formation, cell growth and metabolism^2^,^3^,^4^,^5^,^6^,^7^,^8^. All known deadenylases are magnesium-dependent and belong to one of two superfamilies: The DEDD (Asp-Glu-Asp-Asp) superfamily contains POP2 (or CAF1), CAF1Z, PARN and PAN2 families, whereas the second superfamily includes members related to a class of Exonucleases, Endonucleases, and Phosphatases, known as the EEP (or CCR4) superfamily that includes CCR4, NOCTURNIN, ANGEL, 2′PDE^2^,^9^. It still remains unclear, however, why there are so many deadenylases, whether they are functionally redundant or have distinct roles, and whether these deadenylases act on specific target mRNAs. As the deadenylation process generally occurs in a biphasic manner in mammals, in which long poly(A) tails are first gradually shortened by the PAN2-PAN3 complex to ~100 nt, followed by the CCR4-CAF1-NOT complex to 8–12 nt^10^, it is possible that multiple deadenylases act on the same mRNA with discrete but overlapping functions. However, given the difference in temporal and spatial expression patterns of the deadenylases^5^,^8^,^10^,^11^ and the different phenotypes caused by disrupting specific deadenylases^3^,^4^,^5^,^6^,^7^, it is more likely that each deadenylase targets a specific set of transcripts, although this has not been clearly demonstrated.

Among these deadenylases, *Noc* (Gene name; *Ccrn4l*) is unique, in that it exhibits a robustly rhythmic expression pattern driven by the biological clock, peaking during the night with particularly high amplitude rhythms in liver^12^,^13^. *Noc* is also unique in that it is an immediate early gene (IEG), showing acute responses to several stimuli including serum shock, phorbol ester, lipopolysaccharide (LPS) and rosiglitazone, a peroxisome proliferator-activated receptor γ (PPARγ) agonist^14^,^15^,^16^. NOC has a conserved catalytic domain in the C-terminus, with sequence similarities to other CCR4 family members, but it has a significantly divergent N-terminus and lacks the leucine-rich repeat region required for yeast Ccr4p and mammalian CCR4a and CCR4b to interact with Caf1 and other proteins in the major CCR4–NOT complex^10^,^11^,^17^. These differences in expression pattern and structure suggest that NOC has a function distinct from other members of this protein family.

Mice lacking *Noc* (*Noc* KO) are resistant to diet-induced obesity and hepatic steatosis, yet do not have reduced food intake, increased activity, or measureable changes in whole body energy expenditure when fed a High-Fat Diet (HFD)^3^. This phenotype is due, at least in part, to abnormal dietary lipid trafficking in intestinal enterocytes, preventing efficient energy intake from diet^18^. *Noc* is also one of the differentiation switches of mesenchymal stromal cells (MSCs), supporting a shift towards adipogenesis rather than osteogenesis^15^,^19^. However it is unknown whether the deadenylase function of NOC contributes to these phenotypes, and if so, which specific transcripts NOC targets for its deadenylase activity.

Because *Noc* exhibits a unique expression pattern that is different from other deadenylases^8^,^16^, we hypothesized that NOC would exert its enzymatic activity on a specific set of transcripts and shorten their poly(A) tail length, and a lack of such regulation would ultimately lead to the phenotypes observed in *Noc* KO mice. In order to attempt to identify these transcripts, we used our recently developed technique, a genome-wide screen which we call “Poly(A)denylome” analysis to assess the poly(A) tail length of mRNAs from WT and *Noc* KO livers in an unbiased manner. Using this analysis, we identified 213 transcripts that have longer poly(A) tail lengths in *Noc* KO mouse liver. Despite *Noc’s* robust rhythmicity, however, the great majority of these transcripts do not exhibit rhythmicity in poly(A) tail length or in their steady-state mRNA level in mouse liver. Instead, these transcripts share surprising yet interesting characteristics: they are generally short in length and have relatively long half-lives. Gene ontology analysis of these transcripts, as well as other mRNAs that have altered abundance in the *Noc* KO (both up- and down-regulated), suggests that NOC-regulated transcripts are enriched in those involved in ribosome functions and in oxidative phosphorylation. Most of the transcripts that have altered tail length in the *Noc* KO livers do not fit our expectations for direct targets of NOC and this suggests that NOC acts, at least in part, through a novel activity or via indirect control of target mRNA metabolism that ultimately contribute to the phenotypes observed in *Noc* KO mice.

## Results and Discussion

### Identification of transcripts that have different poly(A) tail lengths in Noc KO liver

**Figure 1:**
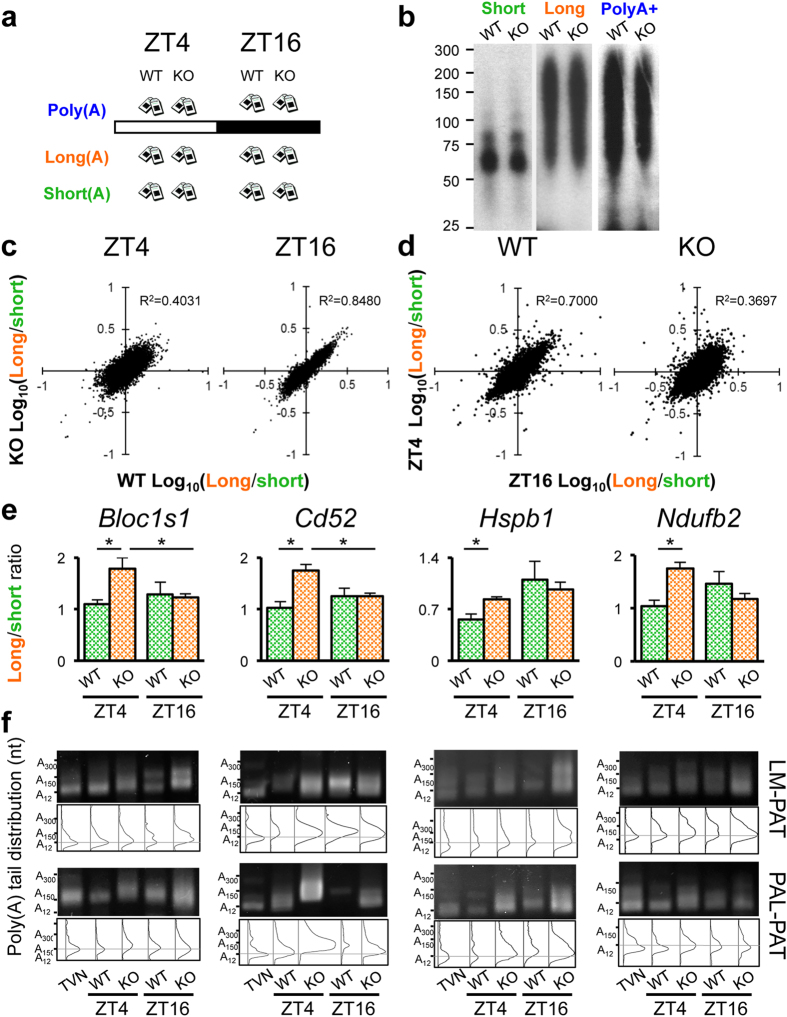
Poly(A)denylome analysis to identify target mRNAs of NOC. (**a**) Shown is the tissue sampling scheme in which mouse livers were harvested at ZT 4 and ZT 16 under 12:12 light:dark (12L:12D) conditions from both WT and *Noc* KO mice (n = 3 per time point). (**b**) The bulk poly(A) tail length (nt) of each fraction was tested by 3′-end labeling assay. Representative gel images are shown. Oligo(dT) chromatography was used to separate total RNA into fractions with either short or long poly(A) tails by varying salt concentrations in the elution^8^. (**c**,**d**) The correlation of the long/short ratio between genotypes (**c**) and time points (**d**). (**e**) Validation of transcripts that have differences in their poly(A) tail length between WT and *Noc* KO. Shown are long-short ratios (**e**) and direct measurements of tail-length by LM-PAT or PAL-PAT assay (**f**). Representative gel images are shown on top and poly(A) tail length distribution calculated by Image J is shown on the bottom. TVN is an oligonucleotide that predominantly recognizes A_12_, serving as an internal control. The horizontal lines across distribution plot depict the expected location of poly(A)_12_. Each lane on gel consists of pooled samples (n = 3 for each time point). All the graphs represent mean ± SEM, *p < 0.05 (Student’s t-test).

In order to identify mRNAs that have altered poly(A) tail lengths in the *Noc* KO mice, we performed “poly(A)denylome” analysis^8^, a method that can measure poly(A) tail length of transcripts in an unbiased and global manner. Mouse livers were collected at Zeitgeber Time 4 (ZT 4; where ZT 0 is defined as time (hours) of lights on and ZT 12 is defined as time of lights off) and ZT 16 (Fig. 1a), time points when the NOC expression is at its nadir and peak, respectively (Supplemental Fig. S2)^12^,^13^. RNAs were then extracted and fractionated by oligo(dT) chromatography into populations that contained either short (<75 nt) or long (60–250 nt) poly(A) tails by varying the salt concentrations in the elution step^8^,^20^ (Fig. 1b). Non-fractionated mRNAs (the total poly(A)^+^ pools) were also obtained as a reference. These RNA samples were subjected to microarray analysis using the Affymetrix Gene ST 1.0 chips that do not require oligo(dT)-based sample preparation to avoid bias from the different poly(A) lengths in the different fractionated samples. Since the correlation of gene expression between each RNA pool was quite strong (Supplemental Fig. 1a–d)^8^, we used the long/short ratio from the microarray analyses as an indicator of poly(A) tail length. Indeed, this normalization largely eliminated the correlation of the expression levels in each fraction (Supplemental Fig. 1d), and we have previously shown that this ratio provides reliable quantitative information on the relative poly(A) tail length^8^.

As was previously reported, there are dynamic circadian changes in poly(A) tail length in WT mouse liver^8^. Since NOC expression is higher during the night (i.e. ZT 16) compared to the daytime (i.e. ZT 4), we expected that the effect of NOC would be stronger and the number of transcripts with longer poly(A) tail in *Noc* KO would be higher at ZT 16. However, the correlation of the long/short ratio between WT and *Noc* KO was weaker at ZT 4 than at ZT 16, indicating there is more variability between two genotypes at ZT 4 (Fig. 1c). We also observed that the long/short ratio was more variable between the two time points in *Noc* KO than in WT (Fig. 1d), despite the known circadian rhythms in tail length in the WT^8^.

In order to identify potential NOC target transcripts, we compared the long/short ratio between WT and *Noc* KO samples and identified transcripts that had significantly different ratios (i.e. tail lengths) in the absence of NOC. Using stringent criteria in which we only examined transcripts that had raw expression values of greater than 200 and a fold-change ratio of greater than 1.5, we identified 309 transcripts that exhibited a statistically significant difference (p < 0.05) at ZT 4, and 10 transcripts at ZT 16 (Supplementary Table 1). All 10 transcripts that were found to differ at ZT 16 had longer tails (higher long/short ratios) in the *Noc* KO samples, while at ZT 4, 203 transcripts had longer tails and 106 transcripts had shorter tails in the *Noc* KO liver. Although it remains unclear why these 106 transcripts had shorter poly(A) tails in *Noc* KO liver, it may be that other deadenylases (all expressed in liver^8^) over-compensate for the lack of *Noc*. It is likely that these differences underestimate the number of transcripts that have altered poly(A) tail length in the KO due to the stringent criteria we used to call a difference in the long/short ratio. In addition, our method does not capture mRNAs with extremely short tails, or lacking tails altogether, which may represent a significant population of mRNAs^20^,^21^,^22^,^23^,^24^.

In order to independently validate the microarray results, the poly(A) tail lengths of randomly chosen transcripts were directly measured using the ligation-mediated poly(A) tail length (LM-PAT) assay^25^ as well as Poly(A) Length (PAL) assay^26^ (Fig. 1e,f). These assays revealed that the poly(A) tail length of the mRNAs we tested indeed exhibited changes between WT and *Noc* KO, as predicted from microarray analyses (Fig. 1e). Due to the heterogeneous nature of poly(A) tail lengths, signals are often detected as smears, therefore, the distribution of sizes detected by densitometry analysis was used to compare the poly(A) tail length between genotypes (Fig. 1f). In addition, these PAT assays also displayed multiple differently-sized PCR products for some mRNAs (such as *Bloc1s1* or *Hspb1* in Fig. 1f), either possibly arising from alternative polyadenylation which occurs in more than 70% of all genes^27^ or representing two populations of transcripts: newly synthesized RNAs with long poly(A) tails and old RNAs that have shortened poly(A) tails.

### Changes in poly(A) tail length do not correlate with changes in mRNA abundance

**Figure 2:**
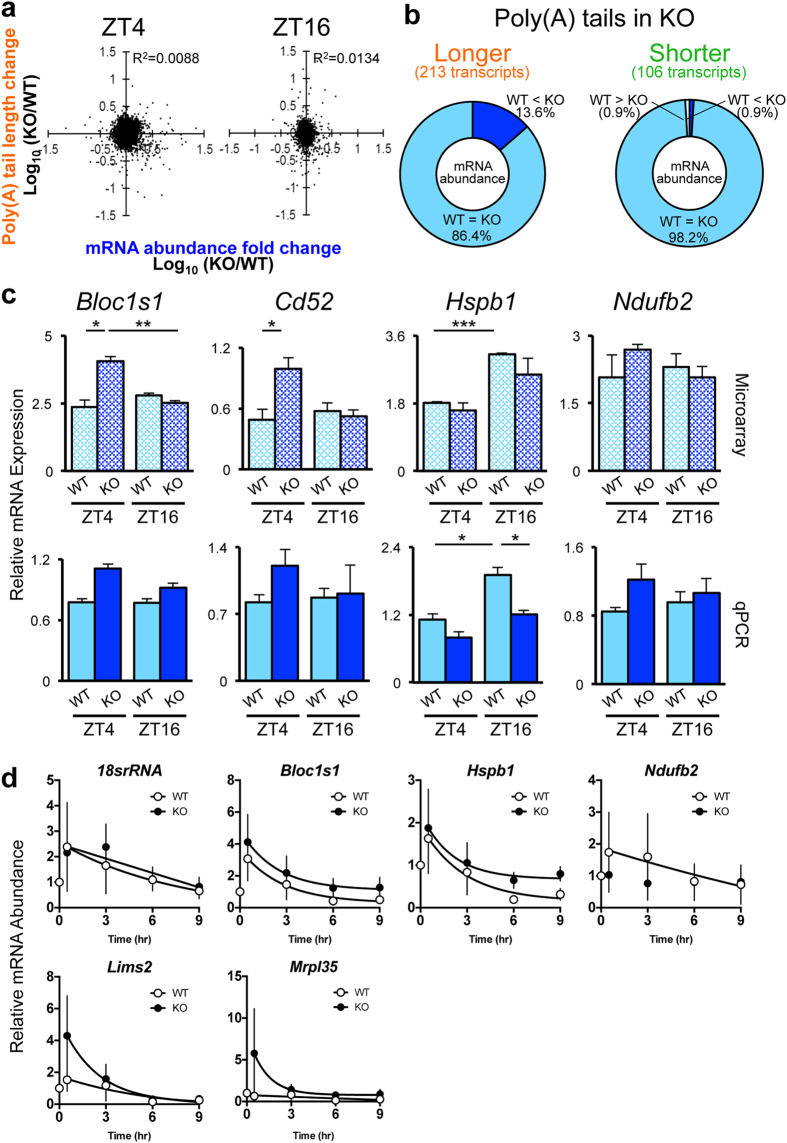
Changes in the poly(A) tail length do not correlate with changes in mRNA abundance. (**a**) Correlation of changes in the long/short ratio vs mRNA abundance between WT and *Noc* KO at each time point. The degree of correlation is shown in the upper right hand corner of each graph. (**b**) Changes in mRNA abundance for transcripts that had longer (left) or shorter (right) poly(A) tail length in *Noc* KO. (**c**) Changes in mRNA abundance of the same four candidate transcripts whose poly(A) tail length difference between WT and *Noc* KO were validated in Fig. 1. mRNA abundance was analyzed by microarray (upper) and qPCR normalized by the expression of *Rplp0 (36B4)* (lower). N = 3 for each sample. All the graphs represent mean ± SEM, *p < 0.05, **p < 0.01, ***p < 0.005 (Student’s t-test). (**d**) mRNA stability in WT and *Noc* KO MEFs. MEFs were treated with 5 ug/ml Actinomycin D for 0, 0.5, 3, 6, 9 hrs and cells were harvested at each time point. The expression of each transcript was measured by qPCR. The expression level of time 0 was set to 1, and all the other data were normalized accordingly. Graphs represent mean ± SEM (n = 3–4).

Because the poly(A) tail length is an important factor in determining the translation initiation and mRNA stability, it has long been thought that depletion of deadenylases would lead to the stabilization of mRNAs, hence increased mRNA abundance^1^. Recent studies, however, have challenged this idea and shown that the loss of a deadenylase can have only small effects on changes in mRNA abundance, and changes in mRNA stability have an inverse correlation with mRNA abundance^28^,^29^. In addition, there is accumulating evidence that mRNA decay and transcription are coupled to buffer mRNA abundances and to maintain RNA homeostasis, and increased mRNA stability is not predictive of increased abundance^30^,^31^,^32^. Because “mRNA abundance” (i.e. the steady-state mRNA levels) represents the sum of RNA degradation and de novo RNA synthesis, longer half-lives may not always result in higher mRNA abundance. We therefore tested whether this is also true in *Noc* KO mouse livers by analyzing the changes in mRNA abundance, obtained from the poly(A)^+^ (non-fractionated) pool and comparing this with changes in poly(A) tail length between WT and *Noc* KO. Interestingly, we did not observe any correlation at either time point (Fig. 2a), supporting the idea that these NOC-dependent changes in poly(A) tail length do not necessarily correlate with the changes in mRNA abundance.

Since we expected NOC targets to have increased tail lengths in the KO, we also examined the relationship between mRNA abundance and poly(A) tail length specifically among the 213 transcripts (from both time points) that had longer poly(A) tail length in *Noc* KO liver. We found that 29 of these transcripts (13.6%) had higher mRNA abundance, but all other transcripts (86.4%) had no changes in mRNA abundance in the *Noc* KO samples. Among the 106 transcripts that had shorter poly(A) tails in *Noc* KO, 1 transcript (0.9%) had lower mRNA abundance and another transcript (0.9%) had higher mRNA abundance in *Noc* KO, while all other transcripts (98.2%) were present at equal levels in both genotypes (Fig. 2B). The changes of mRNA abundance from the microarray data was further confirmed by qPCR for several transcripts that had altered poly(A) tail length (Fig. 2c). In addition to confirming the general lack of correlation between mRNA abundance and poly(A) tail length, in some cases even small differences that had been observed in abundance in the microarrays (i.e. *Bloc1s1* and *Cd52*), did not reach the level of significance when re-examined by quantitative RT-PCR (Fig. 2c). These results suggest that tail shortening by NOC does not usually result in changes in mRNA abundance, but may have some other function.

We also measured the mRNA stability of several transcripts in mouse embryonic fibroblasts (MEFs) derived from both WT and *Noc* KO mice. We observed small changes in half-life in some of the mRNAs that we tested, but for most of the mRNAs we examined, there was no difference in half-life in the *Noc* KO MEFs (Fig. 2d).

### Characteristics of transcripts that have longer poly(A) tails in the Noc KO

The 213 transcripts whose poly(A) tail length (i.e. long/short ratio) was longer in *Noc* KO were of particular interest, because these transcripts fit the pattern expected for bona fide target transcripts of NOC. Therefore, we further hypothesized that NOC target transcripts would share common characteristics that would distinguish themselves as targets. In general, the fate of an mRNA is largely determined by the composition and timing of the interaction between trans-acting factors (i.e. miRNAs and RNA-binding proteins) and cis-elements commonly found in 5′ or 3′ untranslated regions (UTRs) of mRNAs. Therefore, we first attempted to identify cis-elements commonly found in either UTR of the 213 mRNAs that had longer tails in the KOs. However, there was no enrichment of any specific RNA sequence found by HOMER RNA Motif Analysis (http://homer.salk.edu/homer/motif/rnaMotifs.html) (data not shown), although this does not rule out the possibility that NOC recognizes a cis motif defined by RNA secondary structure, rather than RNA sequence.

**Figure 3:**
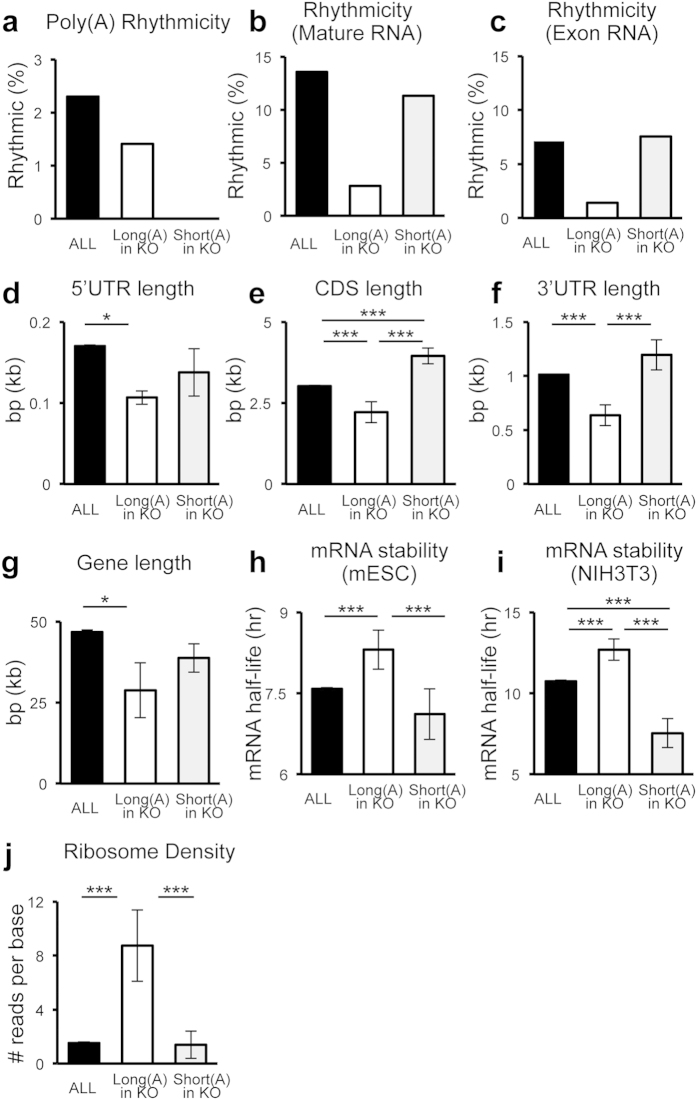
mRNAs with longer tails in the *Noc* KO are short in length, have longer mRNA half-lives, and are actively translated. (**a**) Poly(A) rhythmicity (%)^8^ (ALL; n = 10217, Long(A); n = 148, Short(A); n = 95), (**b**,**c**) Percentage of transcripts that were shown to be rhythmic from circadian transcriptome analyses in mouse liver. (**b**) mature RNA expression^34^ (ALL; n = 20069, Long(A); n = 213, Short(A); n = 106), and **c**) exon expression^33^ (ALL; n = 28661, Long(A); n = 213, Short(A); n = 106). (**d–g**) The length of 5′UTR (**d**) (ALL; n = 33560, Long(A); n = 114, Short(A); n = 77), CDS (**e**) (ALL; n = 29620, Long(A); n = 119, Short(A); n = 78), 3′UTR (**f**) (ALL; n = 33562, Long(A); n = 116, Short(A); n = 80), and gene (**g**) (ALL; n = 31118, Long(A); n = 127, Short(A); n = 78). (**h-i**) mRNA half-lives (hr) in mESCs^35^ (**h**) (ALL; n = 15717, Long(A); n = 93, Short(A); n = 63) and NIH3T3 cells^32^ (**i**) (ALL; n = 4356, Long(A); n = 33, Short(A); n = 16). (**j**) Ribosome footprint density as indicated by the numbers of transcripts bound to polysome fractions in mESCs^36^ (ALL; n = 20055, Long(A); n = 118, Short(A); n = 74). All the graphs represent mean ± SEM, *p < 0.05, **p < 0.01, ***p < 0.005 (Student’s t-test). All analysis was done on 213 transcripts pooled from both the ZT 4 and ZT 16 time points.

Because we have previously shown that many mRNAs have poly(A) tail lengths that change over the daily cycle and because *Noc* is expressed with robust rhythms in mouse liver, we hypothesized that NOC might contribute to this circadian control of poly(A) tail length and therefore NOC targets would be enriched for transcripts with rhythmic poly(A) tails. However, this does not appear to be the case, as only 3 (1.4%) of the 213 putative NOC targets (*Lims2* (peak ZT 16.4), *Alkbh7* (ZT 8.6), *Hspb1* (ZT 16.2) were found to have rhythmic poly(A) tails in our previous analysis^8^, a significant underrepresentation compared to all mRNAs, of which 2.3% had rhythmic poly(A) tail lengths (Fig. 3a). None of the transcripts with shorter poly(A) tail length in *Noc* KO had rhythmic poly(A) tails (Fig. 3a).

In addition, we had originally expected that NOC’s rhythmic expression pattern would contribute to rhythmic mRNA profiles by destabilizing target transcripts during the night. However, this was not the case, because only 3^33^ (*Alkbh7* (peak ZT 21.8), *Safb* (ZT 21.7), *Hist1h1c* (ZT 22.1)) or 6^34^ (*Saa1* (peak ZT1.4), *Hist1h1c* (ZT 0.8), *Arid3a* (ZT 22.4), *Cd52* (ZT 6.4), *Dct* (ZT 3.2), *Hspb1* (ZT 20.8)) of the 213 transcripts were determined to be rhythmic in abundance, based on the two recent datasets of extensively analyzed circadian mRNA profiles at the steady-state level in mouse liver^33^,^34^, a much smaller percent than the percent of total rhythmic mRNAs (Fig. 3b,c).

A recent study also made an interesting observation that the intrinsic poly(A) tail length does not correlate with open reading frame (ORF), untranslated region (UTR) and mRNA length in mouse liver^26^, although in yeast there is a correlation between poly(A) tail length and UTR, mRNA, and ORF length^21^. In order to gain insight into whether this group of mRNAs with extended tails in the *Noc* KO show any such correlation in gene/transcript length, we analyzed the length of 5′- or 3′-UTRs, ORF, and the entire gene. Interestingly, all these parameters were significantly shorter in the transcripts that had longer poly(A) tails in *Noc* KO, as compared to all the transcripts included in the dataset (ALL) (Fig. 3d–g).

It has long been thought that one of the major roles of poly(A) tails is to determine mRNA stability^1^. Therefore, despite the lack of correlation with overall abundance, we wondered whether the mRNAs with longer tails in the *Noc* KO were generally long- or short-lived mRNAs. To this end, we utilized genome-wide datasets of mRNA half-life measurements from mouse embryonic stem cells (mESCs) and NIH3T3 cells^32^,^35^, and examined reported half-lives of this group of mRNAs. Our in silico analyses using these datasets revealed that the transcripts with longer tails in the *Noc* KO have an average half-live that was significantly longer than the average half-live of both the entire group of all mRNAs and those with shorter poly(A) tails in *Noc* KO. This was true for both the mESCs and NIH3T3 cells (Fig. 3h,i).

Another important function of poly(A) tail length is to regulate translation initiation, as mRNAs form a “closed-loop” circular structure in order to initiate translation and the presence of longer poly(A) tails facilitates the formation of this structure^1^, although this relationship has recently been challenged in some situations^26^. Thus, in order to clarify whether NOC may function in translation, we next examined a ribosomal profiling dataset from mES cells^36^, an indicator of translation status. In this dataset, the *Noc* target transcripts are associated with larger numbers of ribosomes, compared to all transcripts and those with shorter tails in the KO (Fig. 3j), indicating that *Noc* target transcripts may belong to a more actively translated group of mRNAs at least in mES cells.

Since poly(A) tail length impacts translation initiation, and NOC targets are generally translationally active (Fig. 3j), we hypothesize that the NOC-dependent changes may be reflected in translational competence and therefore intrinsic protein levels. Recent proteomic analyses from mouse liver over the circadian cycle have provided datasets on rhythmic protein levels^37^,^38^, but the proteins encoded by most of the 213 candidate NOC targets were not represented in these datasets, likely due to the sensitivity limits of the mass spectroscopy.

Together, these results suggest that NOC acts either directly or indirectly on specific sets of target mRNAs to shorten their poly(A) tails but this tail shortening does not generally affect the RNA stability or overall abundance of these mRNAs, and NOC does not seem to significantly regulate rhythmicity of poly(A) tail length or mRNA abundance. Consistent with this, these target mRNAs are generally quite stable, suggesting that tail-shortening by NOC may not be important for regulating mRNA half-life. Nevertheless, it still remains a possibility that NOC regulates mRNA stability, regardless of the changes in poly(A) tail length, until direct NOC-targets can be identified. It should be noted that we have previously reported that NOC stabilizes the iNOS mRNA although it is still unclear whether this is a direct effect^14^.

### Nocturnin’s role in ribosome and mitochondrial oxidative phosphorylation

**Figure 4:**
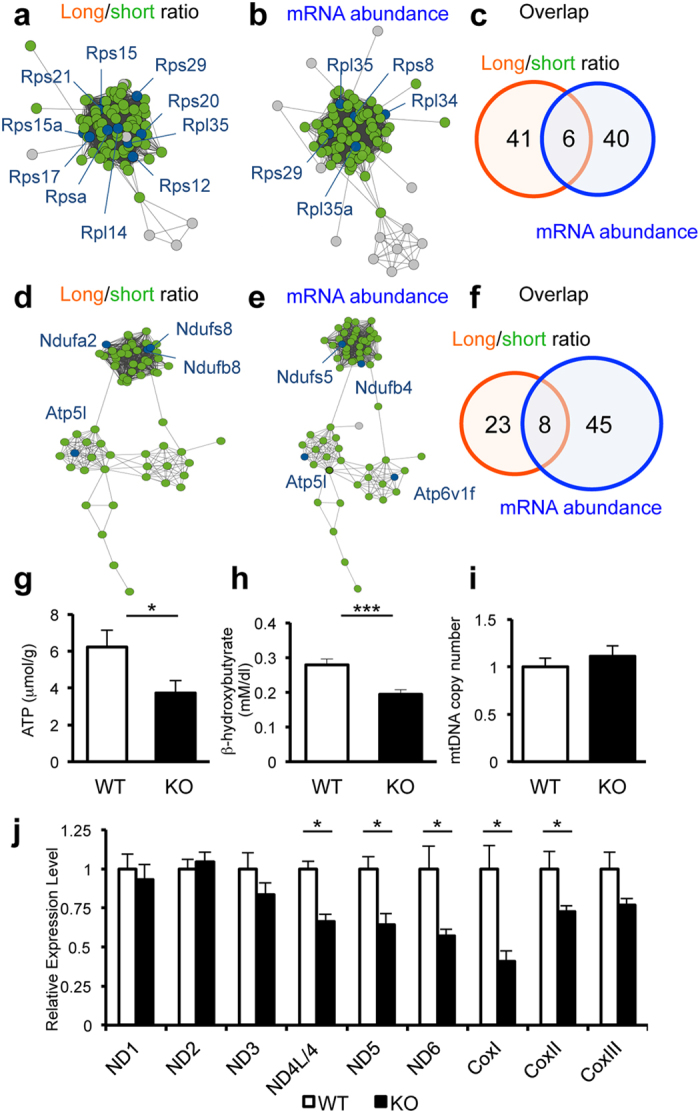
Gene ontology analyses extracted “ribosome” and “oxidative phosphorylation” as biological pathways affected by a loss of NOC. (**a,d**) Transcripts that have longer poly(A) tail (i.e. higher long/short ratio) involved in ribosome cluster (DAVID: p < 0.001, JEPETTO: q < 0.001) (**a**) and oxidative phosphorylation cluster (DAVID: p < 0.05, JEPETTO: q = 0.234) (**d**). Transcripts that had altered mRNA abundance involved in ribosome cluster (DAVID: p < 0.01, JEPETTO: p = 0.084) (**b**), and oxidative phosphorylation cluster (DAVID: p < 0.01, JEPETTO: p = 0.106) (**e**). Data visualization was performed with JEPETTO^41^. Blue nodes represent genes overlapped between target dataset and each process, while green nodes indicate genes specific to each process and grey nodes depict target dataset specific genes. Venn diagrams on the right (**c**,**f**) denote the numbers of transcripts extracted by DAVID^39^ that are known to function in ribosomes (**c**) or oxidative phosphorylation pathways (**f**). Orange circle represents the number of transcript that had higher long/short ratio in *Noc* KO, while blue circle represents the number of transcript that had altered mRNA abundance in *Noc* KO liver. (**g**) Hepatic ATP levels (WT: n = 14, *Noc* KO: n = 10). (**h**) Serum beta-hydroxybutyrate levels (WT: n = 9, *Noc* KO: n = 9). (**i**) Relative copy number of mitochondrial DNA measured by the ratio of mtDNA (*mtCoI*) and nuclear DNA (*18srRNA*) in liver (WT: n = 18, *Noc* KO: n = 18). (**j**) The mRNA expression of mitochondrial-encoded RNAs in liver measured by qPCR (WT: n = 18, *Noc* KO: n = 18). Expression was normalized by nuclear-encoded *Rplp0* expression and the relative expression level of WT was set as 1. All the graphs represent mean ± SEM, *p < 0.05, ***p < 0.005 (Student’s t-test).

In order to determine whether the transcripts that have longer poly(A) tails in *Noc* KO liver belong to particular biological pathways and functions, we performed gene ontology analyses using DAVID^39^,^40^. Pathways significantly enriched in transcripts with longer poly(A) tail length in *Noc* KO were: Ribosomes, Cytosol, Oxidative Phosphophorylation, and Laminin, and these keywords were not enriched in transcripts with shorter poly(A) tail (Supplemental Table S3). Similar results were also obtained when we used JEPETTO^41^, a different algorithm that enabled visualization of the network analysis data (Fig. 4a,b,d,e).

We also performed gene ontology analyses using transcripts that had altered mRNA abundance in *Noc* KO, independent of whether or not their poly(A) tail length changed, and also found the pathways “Mitochondrion” and “Ribosome” to be significantly enriched, and this enrichment was particularly strong in a set of genes that had increased mRNA abundance in *Noc* KO (Supplemental Table 4). Interestingly, this enrichment of transcripts in ribosomal and mitochondrial functions in both datasets is not due to the same sets of transcripts present in both poly(A) and mRNA abundance datasets. For example, among 31 transcripts (out of a total of 213 transcripts) that have differences in their poly(A) tail length (Supplementary Table 1) and 53 transcripts (out of a total of 196 transcripts) that have altered mRNA abundance (Supplementary Table 2) between the two genotypes that are involved in regulating mitochondrial functions, only 9 transcripts (*Atp5l*, *Bloc1s1*, *Mrps24*, *Mrps28*, *Ndufa2*, *Romo1*, *Tmem256*, *Timm13*, *Uqcc2*, *Uqcr*) were included in both datasets (Fig. 4f). Similarly, among 41 (poly(A) tail length) and 40 (mRNA abundance) transcripts that display functions in ribosome, only 6 transcripts (*Mrps24*, *Mrps28*, *Rpl35*, *Rps29*, *Snrpd2*, *Timm13*) were present in both datasets (Fig. 4c). This suggests that NOC plays a prominent function (direct or indirect) in regulation of these biological processes/pathways, through both poly(A) tail length shortening and via changes in mRNA abundance. Because of NOC’s known role in metabolism, we were particularly interested in a potential role for NOC in mitochondrial function. Therefore, we measured hepatic ATP levels as well as serum beta-hydroxybutyrate levels and found that both are decreased in *Noc* KO (Fig. 4g,h), despite that the total number of mitochondria is unchanged (Fig. 4i). Since the function of oxidative phosphorylation is to produce ATP and beta-hydroxybutyrate predominantly in liver from acetyl-CoA by oxidizing fatty acids, these data suggest that the mitochondrial functions are altered in the *Noc* KO. To support this idea, the levels of *Nd4/4L*, *Nd5*, *Nd6*, *CoxI*, and *CoxII*, all of which are mitochondrial RNAs that encode proteins that are involved in Complex I and Complex IV of oxidative phosphorylation chains, are decreased in *Noc* KO (Fig. 4j), further suggesting a role for NOC in regulating the oxidative phosphorylation pathway.

In conclusion, our Poly(A)denylome analysis identified transcripts that have altered poly(A) tail length in *Noc* KO. A significant portion of these transcripts are related to mitochondrial function as revealed by gene ontology analyses (Supplemental Table 3-4) and may contribute to the metabolic phenotypes observed in the *Noc* KO mice^3^,^42^. However, transcripts that have longer poly(A)tails in *Noc* KO liver are relatively stable, and the majority of these transcripts do not exhibit rhythmicity in poly(A) tail length or in their steady-state mRNA level in mouse liver, even though the expression of *Noc* is robustly rhythmic. Therefore, although the poly(A) tail lengths are altered, these may not be direct target transcripts of NOC deadenylase activity. Further supporting this idea is the finding that the number of transcripts that have longer poly(A) tails is higher at ZT 4 when the expression of *Noc* hits nadir, as compared to ZT 16 that the level of *Noc* reaches its peak. In fact, we have not been able to detect any direct physical interaction between NOC and possible target transcripts, even though we have attempted several different techniques with various materials (i.e. cell lines vs tissues, endogenous vs overexpressed NOC). It may be that because NOC does not have an RNA binding domain outside of its catalytic pocket, it only associates with its targets transiently, making it difficult to trap the targets. Together these findings suggest that NOC may be regulating tail length through an indirect mechanism, or through some novel activity. One possibility is that NOC competes against some other deadenylase activity in a rhythmic manner. We have previously shown that the deadenylase activity of NOC is moderate, compared to that of PARN^16^, therefore, slow or incomplete deadenylation by NOC may block complete deadenylation by another more active deadenylase. It will be of interest to explore whether NOC has a molecular function other than a deadenylase.

## Materials and Methods

### Poly(A)denylome analysis

RNA fractionation and the 3′-end labeling assay were performed as described previously^8^. Briefly, mouse liver total RNAs were isolated and resuspended in PolyATract GTC extraction buffer (Promega). RNAs were then mixed with Biotinylated Oligo(dT) Probe (Promega) in dilution buffer (6xSSC, 10 mM Tris-HCl (pH 7.6), 1mM EDTA, 0.25% SDS, 1% β-mercaptoethanol) and incubated for 10 min at 70 °C. After centrifugation at 12,000 g for 10 min at room temperature, supernatant was mixed with Streptavidin MagneSphere Paramagnetic Particles (Promega) and incubated for 15 min at room temperature while nutating. After three washes with 0.5x SSC at room temperature, short poly(A) RNAs were eluted by 0.075x SSC, and subsequently long poly(A) RNAs were eluted by DEPC-treated water. Alternatively, total Poly(A)^+^ RNAs were eluted by DEPC-treated water immediately after the washing step. For bulk poly(A) tail length analysis, RNAs were 3′-end labeled with [^32^P]-pCp with T4 RNA ligase (EPICENTRE) overnight at 4 °C, followed by RNaseA/T1 (Fermentas) digestion to remove the bodies of the mRNAs. The non-digested poly(A) tails were then resolved by 7.5% denaturing PAGE and detected on X-ray film. Fractionated or non-fractionated RNAs were further purified using RNeasy MinElute Cleanup Kit (QIAGEN) for microarray that was performed at Molecular Biology Core Facilities at Dana-Farber Cancer Institute. For each time point at ZT 4 and ZT 16, three biological replicas from WT and *Noc* KO mouse liver were each hybridized to individual microarray chips (Affymetrix Mouse Gene ST 1.0), according to the manufacturer’s instruction. Raw data were analyzed and normalized by the quantile method through the dChip software. Probe sets were annotated using the Affymetrix annotation file as of February 2009.

### Poly(A) tail length measurement

LM-PAT assays were performed as described previously^8^,^25^ with a slight modification. In brief, 50 ng of poly(A)+ enriched RNAs extracted using PolyATract System 1000 (Promega), were first incubated with 5′-phosphorylated oligo(dT)_15_ in the presence of T4 DNA ligase for 30 min at 42 °C to anneal with poly(A) tails of RNAs, followed by an excess amount of anchor primer with oligo(dT)_12_ (5′-GCGAGCTCCGCGGCCGCGTTTTTTTTTTTT-3′) to anneal at the end of poly(A) tails. This was further incubated for 2 h at 12 °C to complete ligation between oligo(dT)s. These oligo(dT)-annealed RNAs were then subjected to reverse transcription reaction using SuperScript III (Life Technolgoies) for cDNA synthesis.

PAL-PAT assays were performed based on the PAL-seq library construction method previously reported^26^. In brief, 1 ug of poly(A)+ enriched RNAs extracted using PolyATract System 1000 (Promega), were first incubated with an adapter (5′-Phos.AGA TCG GAA GAG CGT CGT GTA GGG AAA GAG TGT AGA CAC ATA C-3′) and a splint (5′-TTC CGA TCT TTT TTT TT-3′) in the presence of RNA ligase 2 (NEB) at 18 °C for overnight to attach the splint adapter at the 3′-end of poly(A)+ RNAs. These adapter-ligated RNAs were then partially digested by RNaseT1 (Life Technologies) for 30 min at room temperature, followed by reverse transcription reaction using SuperScript III (Life Technolgoies) with solexa_rt_primer (5′-AAT GAT ACG GCG ACC ACC GAG ATC TAC ACT CTT TCC CTA CAC G -3′) after inactivating RNaseT1 by Precipitation/inactivation buffer (Life Technologies).

Aliquots of this cDNA were used as templates for PCR reactions with message-specific primers of our mRNAs of interest. Then, PCR products were digested by a restriction enzyme to confirm the specificity. Resulting DNA fragments were visualized by Gel-Doc system (Bio-Rad), and poly(A) tail length distribution was visualized by Image J. Primer sequences and restriction enzymes used in both PAT assays can be found in Table S5.

### Animals, cells, qRT-PCR, and DNA extraction

Male mice of WT and *Noc* KO^3^ were maintained on a 12:12 LD cycle and fed *ad libitum*. All the procedures were performed in accordance with the Guideline of Institutional Animal Care and Use Committee (IACUC) of UT Southwestern Medical Center and were approved by the IACUC of UT Southwestern Medical Center. Mouse liver samples for Poly(A)denylome, qPCR and Western blot analyses were taken from three biological replicas of WT and *Noc* KO at ZT 4 and ZT 16. Immediately after mice were sacrificed, livers were macrodissected and then snap-frozen in liquid nitrogen. Frozen livers were stored at −80C until RNA, DNA, proteins were extracted within one year.

Frozen mouse livers were mechanically homogenized (Kinematica) to extract RNA and protein. Total RNAs were extracted using TRIZOL reagent (Life Technologies), and then poly(A) enriched RNAs were extracted by Poly(A)Tract System (Promega). After the RNA quantification by NanoDrop (Thermo Scientific), 50 ng of poly(A) enriched RNAs were subjected to cDNA synthesis (total volume 20 ul) with Oligo(dT)_12-18_ primer (Life Technologies) using 1 ul/sample of SuperScript II (Life Technologies) according to manufacturer’s instructions. DNase I treatment was not performed at any point. Quantitative PCR (qPCR) was performed using ABI7900 (Applied Biosystems) with SYBR Power Green (Applied Biosystems) with a total volume of 10 ul/well in 384 well plate including 1ul of cDNA (diluted in 1/50 after cDNA synthesis) using relative standard curve method. To quantify each gene expression, a standard curve was generated for each primer set with serial 5-fold dilutions (linear dynamic range 1/10-1/6250) using the WT mouse liver cDNAs, and the limit of detection was empirically determined. Cycling parameters were as follows: holding at 50 °C for 2 min and 95 °C for 10 min, then 40 cycles of 95 °C for 15 sec and 60 °C for 1 min. Subsequently, melting curves were drawn to check the target specificity of each reaction. Primers were designed to flank intron sequences and include all the isoforms whenever possible and their specificity was tested by BLAST. Primer concentrations were determined based on the test run to yield the most reproducible and reliable results at the lowest concentration, and there was no obvious PCR inhibition observed with any primer pair. No primer pairs yielded any signals for Non-Template (DNA) control under our conditions. Primer information used in qPCR analyses can be found in Table S5, and the parameters for standard curves for each gene can be found in Table S6. Data were analyzed by SDS v2.3 (Applied Biosystems) and each Ct was determined automatically. All the data were normalized to the expression level of Rplp0 (36B4), as Rplp0 has been shown to have no fluctuation in the gene expression around the circadian clock in mouse liver^43^, and we did not omit any data as outliers. cDNA synthesis and qPCR was performed once using three biological replicas, all of which were run as duplicates in qPCR.

Nuclear and mitochondrial DNAs were extracted from frozen liver samples using DNeasy Blood and Tissue Kit (QIAGEN) according to the manufacturer’s instructions. Isolated DNAs were subjected to qPCR analysis to quantify the relative mt copy number by measuring the relative amount of nuclear-DNA (18srRNA) and mitochondria-DNA (mtCoxI).

Primary mouse embryonic fibroblasts (MEFs) from both WT and *Noc* KO^14^ were immortalized by introducing aP53 expressing retrovirus^44^. These immortalized MEFs were treated with 5 ul/mg actinomycin D (Sigma) for 0, 0.5, 3, 6, and 9 hrs before RNA was extracted.

### Hepatic ATP and serum beta-hydroxybutyrate measurements

Hepatic ATP concentration was measured as previously described^45^. Briefly, snap frozen aliquots of liver were homogenized in ice-cold DMEM supplemented with 5% perchloric acid. After centrifuging at 16,000 g for 10 min at 4°C, the homogenates were neutralized by 10N NaOH and 1 M Tris-HCl (pH 7.4), ATP concentration was measured using CellTiter-Glo Luminescent Cell Viability Assay (Promega) according to the manufacturer’s instructions. The results were normalized by measuring protein concentration of each sample. Serum beta-hydroxybutyrate concentration was measured by the Mouse Metabolic Phenotyping Centers (National Mouse Metabolic Phenotyping Centers).

## Additional Information

**How to cite this article**: Kojima, S. *et al.* Changes in poly(A) tail length dynamics from the loss of the circadian deadenylase Nocturnin. *Sci. Rep.* **5**, 17059; doi: 10.1038/srep17059 (2015).

## Supplementary Material

Supplementary Information

Supplementary Table S1

Supplementary Table S2

Supplementary Table S3

Supplementary Table S4

Supplementary Table S5
