# Supplementary material for: Changes in poly(A) tail length dynamics from the loss of the circadian deadenylase Nocturnin
Source: Sci Rep. 2015 Nov 20;5:17059. doi: 10.1038/srep17059 (PMC4653638; doi:10.1038/srep17059)
Supplement: Supplementary Information [file srep17059-s1.pdf]

Supplementary Information

Changes in poly(A) tail length dynamics from the loss of the circadian deadenylase Nocturnin

Shihoko Kojima[1, 2, *], Kerry L. Gendreau[2], Elaine L. Sher-Chen[1], Peng Gao[1], Carla B. Green[1]

1. Department of Neuroscience, University of Texas Southwestern Medical Center, Dallas, TX, USA, 75390-9111
2. Department of Biological Sciences, Virginia Bioinformatics Institute, Virginia Tech, Blacksburg, VA, USA, 24060

* Corresponding author
Shihoko Kojima, Ph. D.
Department of Biological Sciences, Virginia Bioinformatics Institute, Virginia Tech
1015 Life Science Circle, 162B
Blacksburg, VA, 24061
Email: skojima@vt.edu
Phone: +1-540-231-5196

Supplemental Figure 1. Kojima et al.,

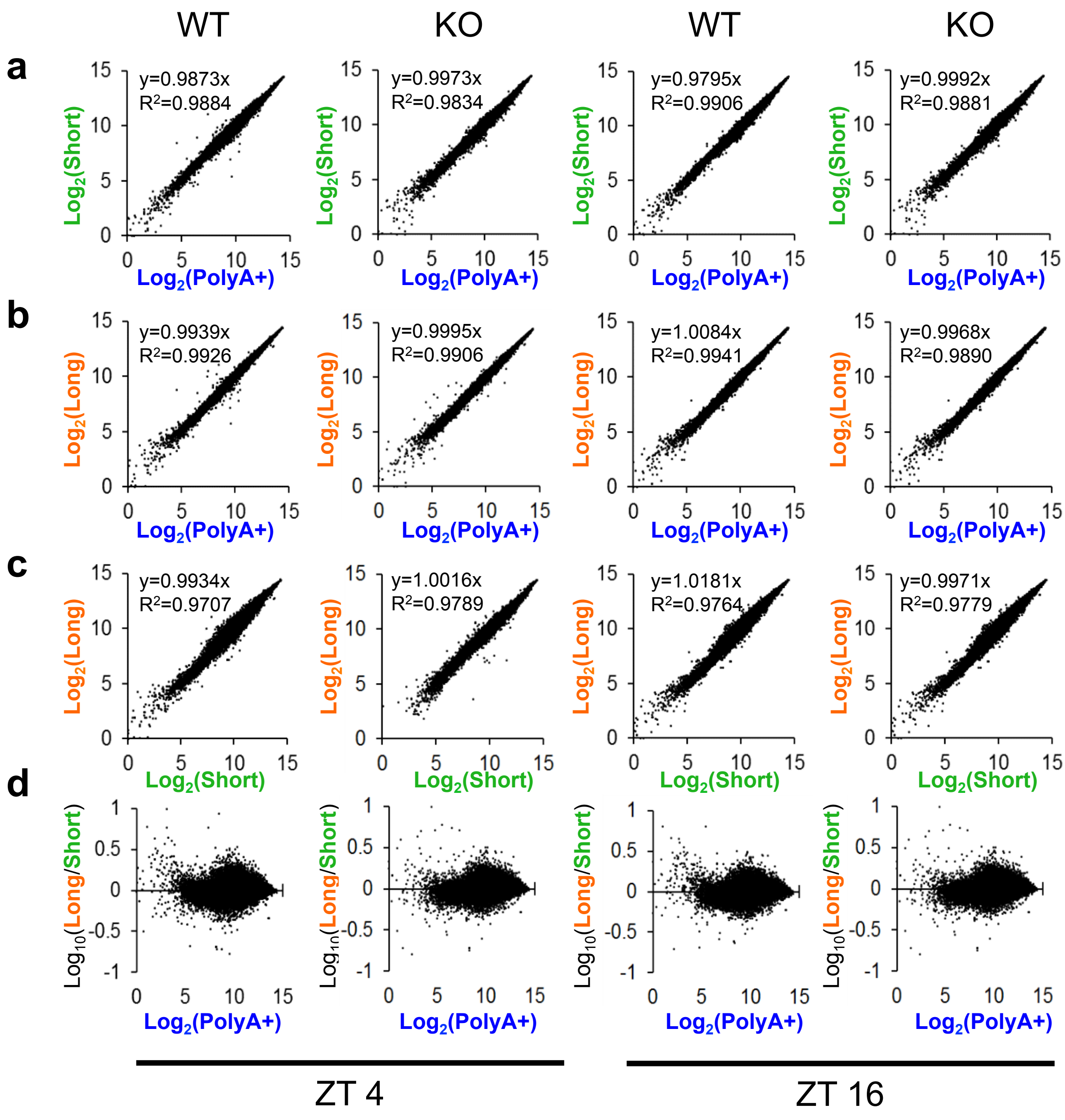

Figure S1. Comparison of the signal intensity of each mRNA in the polyA+ samples versus the short-tailed samples (**A**), in the polyA+ samples versus the long-tailed samples (**B**), in the short-tailed samples versus the long-tailed samples (**C**), and in the polyA+ samples versus the $\log_{10}$(long/short ratio) (**D**). The degree of correlation is shown in the top left corner of each graph (**A**-**C**). The data for WT has been published elsewhere [1].

1. Kojima, S., Sher-Chen, E. L. & Green, C. B. Circadian control of mRNA polyadenylation dynamics regulates rhythmic protein expression. *Genes Dev* **26**, 2724-2736, doi:10.1101/gad.208306.112 (2012).
